# Mechanical contributors to sex differences in idiopathic knee osteoarthritis

**DOI:** 10.1186/2042-6410-3-28

**Published:** 2012-12-23

**Authors:** Daniel P Nicolella, Mary I O’Connor, Roger M Enoka, Barbara D Boyan, David A Hart, Eileen Resnick, Karen J Berkley, Kathleen A Sluka, C Kent Kwoh, Laura L Tosi, Richard D Coutts, Lorena M Havill, Wendy M Kohrt

**Affiliations:** 1 Isis Research Network on Musculoskeletal Health, Society for Women’s Health Research, Washington, DC, 20036, USA; 2 Mechanical Engineering Division, Southwest Research Institute, San Antonio, TX, 78238, USA; 3 Department of Orthopedic Surgery, Mayo Clinic, Jacksonville, FL, 32224, USA; 4 Department of Integrative Physiology, University of Colorado Boulder, Boulder, CO, 80309, USA; 5 Wallace H. Coulter Department of Biomedical Engineering, Georgia Institute of Technology, Atlanta, GA, 30332, USA; 6 Department of Surgery, University of Calgary, Calgary, AB, T2N 4N1, Canada; 7 Program of Neuroscience, Florida State University, Tallahassee, FL, 32306, USA; 8 Department of Physical Therapy and Rehabilitation Science, University of Iowa, Iowa City, IA, 52242, USA; 9 Division of Rheumatology and Clinical Immunology, University of Pittsburgh, Pittsburgh, PA, 15261, USA; 10 Department of Orthopedic Surgery and Pediatrics, George Washington University, Washington, DC, 20010, USA; 11 Department of Orthopedic Surgery, University of California, San Diego, San Diego, CA, 92123, USA; 12 Department of Genetics, Texas Biomedical Research Institute, San Antonio, TX, 78245, USA; 13 Division of Geriatric Medicine, University of Colorado Denver, Aurora, CO, 80045, USA

**Keywords:** Knee joint, Limb alignment, Muscle function, Obesity, Osteoarthritis, Sex differences

## Abstract

The occurrence of knee osteoarthritis (OA) increases with age and is more common in women compared with men, especially after the age of 50 years. Recent work suggests that contact stress in the knee cartilage is a significant predictor of the risk for developing knee OA. Significant gaps in knowledge remain, however, as to how changes in musculoskeletal traits disturb the normal mechanical environment of the knee and contribute to sex differences in the initiation and progression of idiopathic knee OA. To illustrate this knowledge deficit, we summarize what is known about the influence of limb alignment, muscle function, and obesity on sex differences in knee OA. Observational data suggest that limb alignment can predict the development of radiographic signs of knee OA, potentially due to increased stresses and strains within the joint. However, these data do not indicate how limb alignment could contribute to sex differences in either the development or worsening of knee OA. Similarly, the strength of the knee extensor muscles is compromised in women who develop radiographic and symptomatic signs of knee OA, but the extent to which the decline in muscle function precedes the development of the disease is uncertain. Even less is known about how changes in muscle function might contribute to the worsening of knee OA. Conversely, obesity is a stronger predictor of developing knee OA symptoms in women than in men. The influence of obesity on developing knee OA symptoms is not associated with deviation in limb alignment, but BMI predicts the worsening of the symptoms only in individuals with neutral and valgus (knock-kneed) knees. It is more likely, however, that obesity modulates OA through a combination of systemic effects, particularly an increase in inflammatory cytokines, and mechanical factors within the joint. The absence of strong associations of these surrogate measures of the mechanical environment in the knee joint with sex differences in the development and progression of knee OA suggests that a more multifactorial and integrative approach in the study of this disease is needed. We identify gaps in knowledge related to mechanical influences on the sex differences in knee OA.

## Review

As discussed in the preceding introduction to our series of papers, the exact etiology of knee osteoarthritis (OA) is not well understood. One prominent theory on the mechanisms underlying the development of knee OA attributes a primary role to perturbations of the local mechanical environment [1], especially the loads experienced by the articular cartilage. Recent computational studies, for example, have indicated that the risk of developing a combination of symptoms and radiographic signs of knee OA at 15 months after a baseline evaluation can be predicted by estimates of the contact stress on the cartilage during a static standing position [2]. Individual differences in joint mechanics, therefore, are presumed to contribute significantly to the degradation of the cartilage during idiopathic knee OA [3].

Three prominent risk factors for the development and worsening of knee OA are limb alignment, muscle weakness, and obesity, all of which alter the mechanical environment of the joint. However, not all individuals who present with these risk factors develop knee OA and, conversely, many individuals who do not exhibit these risk factors develop OA in later years [4]. Consequently, the hypothesis has emerged that differences in joint mechanics, driven by normal variability in joint anatomy and biological predisposition, underlie much of the variation in risk of knee OA onset and progression. However, there are no effective means of identifying which of those individuals who present with these risk factors will develop knee OA.

Understanding how local mechanical stress in the knee joint contributes to knee OA is key to developing intervention strategies to minimize the development and worsening of the disease. However, it is difficult to measure the mechanical environment of the cartilage *in vivo* and the use of computational methods to estimate cartilage stresses is not practical in clinical settings. Consequently, the typical approach has been to use clinically measureable surrogates, such as musculoskeletal traits, that influence *in vivo* cartilage stress. Significant gaps in knowledge remain as to how sex differences in limb alignment, muscle function, and obesity disturb the normal mechanical environment of the knee and cartilage and thereby underlie sex differences in knee OA. To illustrate this deficit in knowledge, we summarize what is known and not known about how limb alignment, muscle function, and obesity influence sex differences in the development and worsening of knee OA. Other mechanical factors may be associated with the development of knee OA, but these are not addressed in this brief review.

### Limb alignment

**Figure 1:**
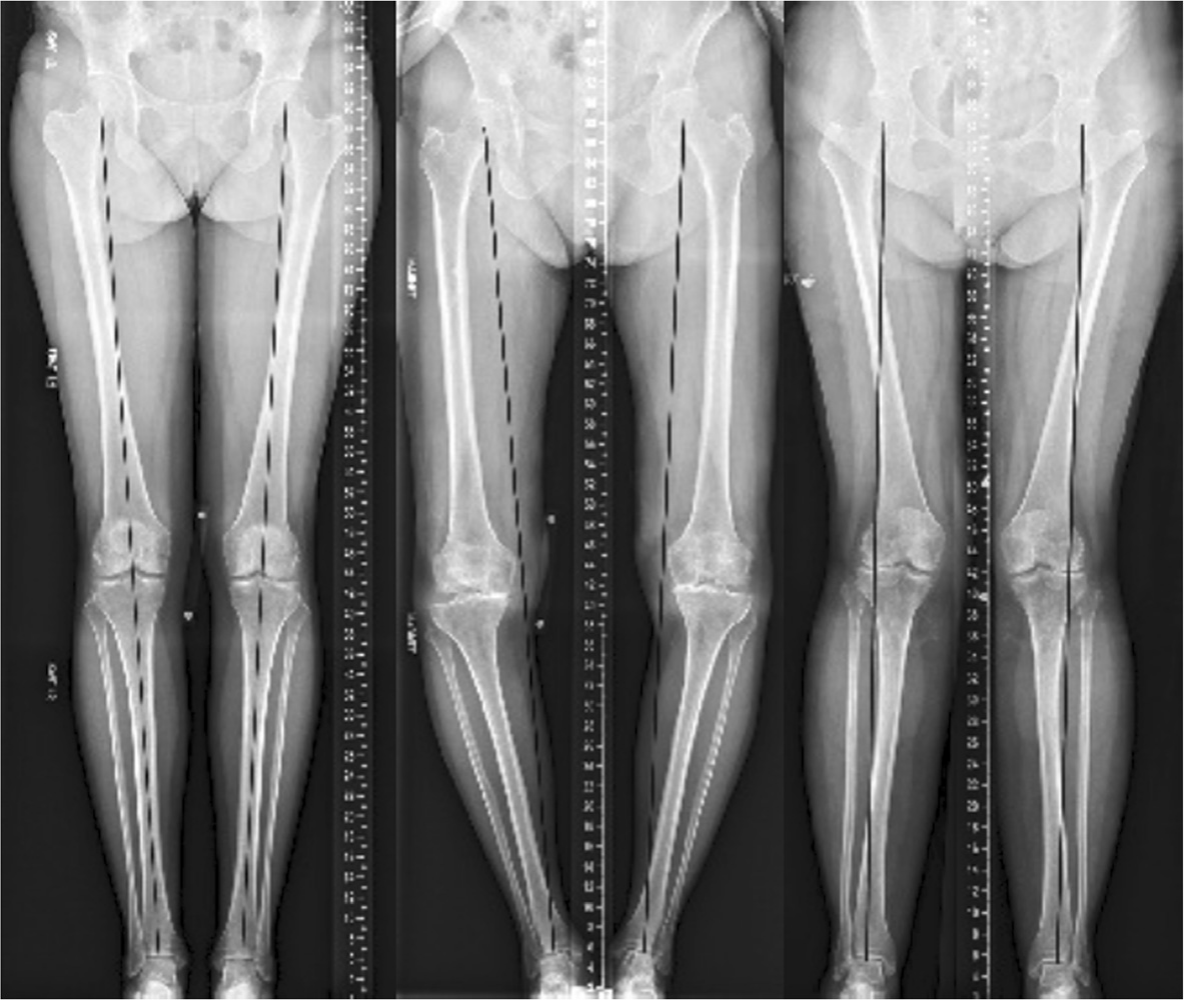
**Full-length radiographs of both lower extremities showing neutral (left), varus (middle), and valgus (right) limb alignment.** A line is drawn on each image from the center of the femoral head (representing the center of the hip joint) to the center of the ankle joint (talus). *Left*. When the line transects the knee joint, as in the neutral alignment, the weight-bearing stresses are well distributed in the lower extremity. *Middle*. When the line is medial to the center of the knee joint (varus alignment), there is an abnormal distribution of weight-bearing stresses on the medial (inner) aspect of the knee joint. *Right*. Conversely, when the line is located lateral to the center of the knee joint (valgus alignment), the weight-bearing stresses are greater on the lateral aspect of the knee joint.

Limb alignment, measured as the hip-knee-ankle angle from a full-length radiograph, is characterized as valgus (knock-kneed), varus (bow-legged), or neutral. Varus limb alignment shifts the center of pressure within the knee medially and increases the external knee adduction moment during gait, which results in a greater proportion of the load being borne by the medial compartment of the contact between the femur and tibia (e.g., medial condyles) [5]. Conversely, valgus limb alignment shifts the center of pressure laterally and reduces the external adduction moment about the knee during gait, which increases lateral-compartment loading (e.g., lateral condyles) (Figure 1). The influence of limb alignment on the distribution of the load in the knee joint, and consequently the local mechanical environment of the cartilage during movement, is presumed to be a significant contributor to the erosion of cartilage, which is a fundamental characteristic of knee OA [6].

Despite the presumed negative influence of deviations in limb alignment from neutral, an observational study of 250 healthy adults (20–27 years) found that 32% of men and 17% of women had varus alignment (mechanical axis alignment of ≥3 degrees from neutral) at skeletal maturity, which has been termed ‘constitutional varus’ [7]. There are no data on how this alignment influences either the development or progression of knee OA. In addition, limb alignment may not be symmetric within an individual, which may lead to unilateral development of knee OA due to differences in knee-joint loading between the left and right limbs. However, sex differences in unilateral vs. bilateral limb alignment and the subsequent role in the risk of incidence and progression of knee OA have not been examined and represent a significant gap in knowledge.

#### Limb alignment and the development of knee OA

Because limb alignment is relatively easy to measure *in vivo*, its relation with knee OA has been examined in a number of studies. In the longitudinal Multicenter Osteoarthritis Study (MOST), an investigation of 2,958 knees *without radiographic evidence of knee OA at baseline* but with frequent symptoms consistent with knee OA or at risk for developing knee OA, found that the risk of developing radiographic evidence of knee OA at 30 months after an initial evaluation was significantly elevated in individuals with varus knee alignment compared with neutral knee alignment (OR 1.49), whereas valgus alignment did not influence the risk of developing knee OA (OR 0.87) [8]. When limb alignment was considered as a continuous variable, the risk of developing knee OA was related to the magnitude of misalignment for varus limbs; a greater severity of varus alignment at baseline was associated with a higher risk of radiographic signs of knee OA. There was no such relation for valgus alignment. Furthermore, there were no sex differences in the risk of developing radiographic evidence of knee OA due to limb alignment. Similarly, varus alignment was associated with a 2-fold increase in risk of developing radiographic signs of knee OA compared with a neutral knee alignment in the Rotterdam study of 1,501 participants (2,664 knees), whereas valgus alignment was associated with a 1.5-fold increase of developing knee OA risk [9]. The results were not influenced by the sex of the participants. Conversely, there was no association between limb alignment and the development of radiographic signs of knee OA over ~9 years in the Framingham cohort, although this study estimated limb alignment from X-rays of the knee and compared the most varus knees with the most valgus knees [10].

#### Limb alignment and the progression of knee OA

When there was *radiographic evidence of knee OA at baseline* in the MOST study, those knees in a limb with varus alignment were at significantly greater risk for a worsening of the radiographic signs in the medial compartment at 30 months (OR 3.59), whereas knees in a limb with valgus alignment at baseline were at greater risk for an increase in the radiographic signs of OA in the lateral compartment at 30 months [8]. There were no sex differences in either association. Conversely, only those participants in the Rotterdam study with varus alignment of the limb experienced a significant increase in risk of the radiographic signs of knee OA becoming worse [9]. These observations were not categorized by either the compartment in which the radiographic evidence was observed or the sex of the participants.

Taken together, these data suggest that limb alignment is a predictor of both the appearance and worsening of radiographic signs of knee OA and that varus and valgus alignment each increase the risk of the radiographic evidence becoming more severe in the compartment that experiences an increase in the load transmitted through the joint. However, the association between limb alignment and cartilage stress is relatively modest because the stress depends on the geometry of the opposing joint surface, the material properties of the tissues (e.g., the stiffness of the ligaments, menisci, cartilage, and subchondral bone), and the forces acting across the knee joint. To examine these associations in more detail, patient-specific predictions of cartilage stress when standing on a single leg were simulated with a finite element model. The simulations indicated that the cartilage stress and strain in the medial compartment was greater for an individual with varus alignment, whereas the cartilage stress and strains in the lateral compartment were greater when the limb exhibited a valgus alignment (Table 1) [11]. The results from this computational study are consistent with the hypothesis that deviation of the limb from a neutral alignment has a direct influence on cartilage stress *in vivo* and can likely contribute to the development of knee OA and the rate at which the disease progresses.

**Table 1:** The influence of limb alignment on the normal stress (relative to body weight) and strain for the medial (top) and the lateral (bottom) cartilage of the tibia and femur during the gait cycle

	**Medial Cartilage Normal Stress**		**Medial Cartilage Normal Strain**	
**Subject**	**Tibia**	**Femur**	**Tibia**	**Femur**
1 (varus)	0.020	0.023	18.66	26.66
2 (normal)	0.017	0.020	17.01	20.67
3 (valgus)	0.016	0.018	14.99	16.16
	**Lateral Cartilage Normal Stress**		**Lateral Cartilage Normal Strain**	
**Subject**	**Tibia**	**Femur**	**Tibia**	**Femur**
1 (varus)	0.001	0.003	4.64	6.79
2 (normal)	0.003	0.004	8.06	11.41
3 (valgus)	0.008	0.010	9.22	11.38

The data were obtained at 25% of the stance phase when both the axial load and the varus knee moment were at peak values. The results showed the magnitude of the stresses and strains in the medial compartment increased with varus alignment, whereas the magnitude of the stresses and strains in the lateral compartment increased with valgus alignment [61].

Despite a probable role for limb alignment in contributing to the initiation and progression of knee OA, sex differences in lower limb alignment are equivocal (Table 2) [5,12-16]. Some of this uncertainty can be attributed to differences across studies in the methods used to assess limb alignment [17], the confounding influence of ethnicity on limb alignment, changes in limb alignment across the lifespan, and the relation of limb alignment during standing with such dynamic actions as walking. In summary, there is no obvious sex difference in the influence of limb alignment on knee OA among older adults, despite the greater prevalence of knee OA among women in this age group [5,16]. We have identified several gaps in knowledge related to the influence of limb alignment on the sex differences in knee OA (Table 3).

**Table 2:** Distributions of limb alignment by age, sex, and ethnicity

**Limb alignment measure**	**Ethnicity**	**Sex**	**Age (years)**	**Alignment Mean±SD**	**Source Note/conclusion**
Femur-tibia angle (FTA) (degrees, mean ± SD)	Not specified	men	21-40 (n=30)	2.3 ± 2.3 varus	[5] No age or sex differences Mean FTA 1.2^o^ ± 2.2^o^ (varus)
			41-60 (n=30)	1.0 ± 2.3 varus	
		women	21-40 (n=30)	1.3 ± 1.8 varus	
			41-60 (n=30)	0.3 ± 2.3 varus	
FTA (degrees, mean ± SD)	Chinese	men	mean age: 24 range: 22–31 (n=25)	2.2 ± 2.7 varus	[15] No sex differences
		women	mean age: 23 range: 21–29 (n=25)	2.2 ± 2.5 varus	
FTA (degrees, mean ± SD)	Japanese and Australian Caucasian	men	18-29 (n=21)	180.3 ± 3.0 varus	[16] In combined group, women had more valgus alignment p = 0.017) Japanese (men and women) more varus than Australian Caucasians; No age effects
			30-59 (n=36)	179.8 ± 2.5 valgus	
			>60 (n=23)	180.0 ± 2.1 neutral	
		women	18-29 (n=35)	179.5 ± 3.2, valgus	
			30-59 (n=36)	178.6 ± 2.5 valgus	
			>60 (n=23)	180.0 ± 2.1 neutral	
Hip-knee-Ankle (HKA) (degrees, mean ± SD)	Not specified/Canadian	men	<30 (n=38) >45 (n=14)	−1.5 ± 3.0 varus	[13] Women more likely than men in all age groups to have valgus alignment (p = 0.03) No age effects
		women	<30 (n=41) >45 (n=26)	−0.5 ± 2.6 varus	
HKA	Japanese and Caucasian	men	Caucasian 28 ± 6.8 (n=23)	36% of men had valgus alignment	[14] Japanese higher varus vs. Caucasians; Women more valgus than men
			Japanese 30 ± 6.3 (n=11)		
		women	Caucasian 26 ± 7.7 (n=24)	50% women had valgus alignment	
			Japanese 37 ± 6.2 (n=12)		

### Muscle function

Because the forces exerted by the muscles in the entire limb contribute to the contact forces experienced by the tissues that comprise the knee joint [17], questions arise regarding the extent to which changes in muscle func-tion can modify the integrity of the knee joint and whether there are differences between men and women.

#### Muscle strength as a predictor of the development of knee OA

**Figure 2:**
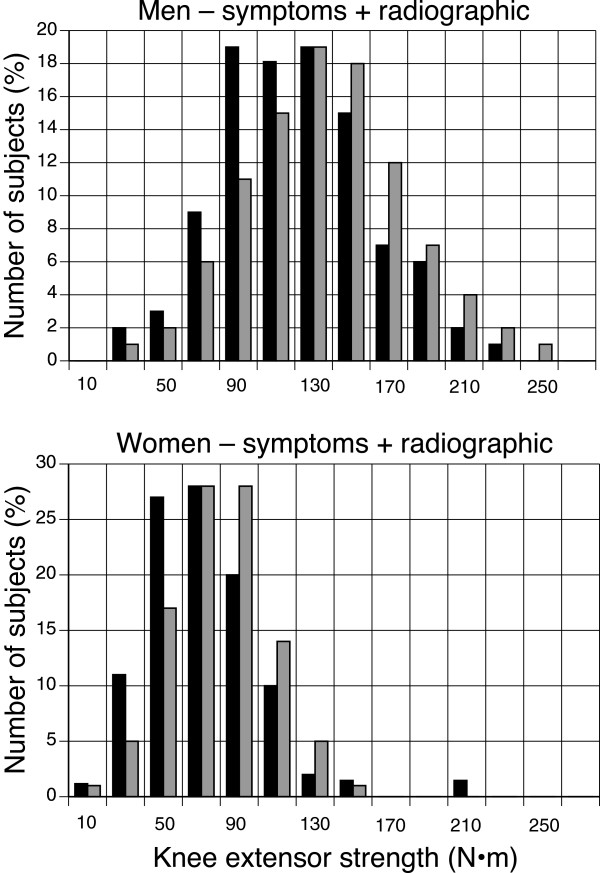
**Associations between knee extensor strength and the incidence of developing *radiographic *signs of tibiofemoral OA at 30 months of follow-up **[20]**.** Those men (n = 70) and women (n = 198) in the MOST study who exhibited the radiographic signs are indicated in black bars and those who did not (men: 1,110; women: 2,679) are shown in grey bars. The data are plotted as percentages of the number of subjects in each group. Strength was measured on an isokinetic dynamometer using shortening contractions performed at 60º/s. Data were provided by Neil A. Segal, M.D.

Cross-sectional studies suggest that weakness of the knee extensor muscles may precede the development of knee OA [18,19]. However, results from prospective cohort studies that assessed whether quadriceps strength predicts incident knee OA are equivocal. The longitudinal Multicenter Osteoarthritis Study (MOST) of 1,617 participants (2,519 knees) who did not exhibit radiographic signs of tibiofemoral OA at baseline found that neither knee extensor strength nor the relative strength of the hamstring muscles predicted incident *radiographic* (i.e., asymptomatic) evidence for tibiofemoral OA (48/680 men, 49 knees; 93/937 women, 99 knees) 30 months later [20]. The knee extensor strength for men and women who developed *radiographic* tibiofemoral OA (mean ± SD) was 123 ± 48 and 74 ± 29 N·m, respectively, compared with 131 ± 43 and 76 ± 25 N·m for those who did not (Figure 2). When women and men were grouped by tertiles of knee extensor strength, the odds ratios for incident *radiographic* OA in women and men with the highest strength levels (compared with the lowest) were 0.86 (CI: 0.65,1.14) and 0.76 (CI: 0.52,1.11), respectively.

**Figure 3:**
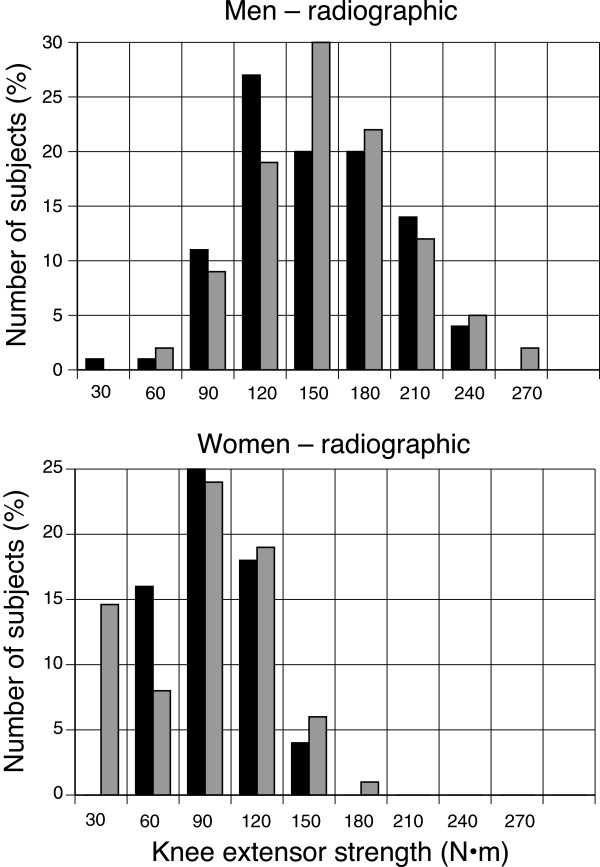
**Associations between knee extensor strength and the incidence of developing *symptomatic *and *radiographic *signs of knee OA at 30 months of follow-up **[20]**.** Those men (n = 101) and women (n = 217) in the MOST study who exhibited both signs of knee OA are indicated in black bars and those who did not (men: 1,535; women: 2,223) are shown in grey bars. The data are plotted as percentages of the number of subjects in each group. Strength was measured on an isokinetic dynamometer using shortening contractions performed at 60º/s. Data were provided by Neil A. Segal, M.D.

In contrast, the same study found that knee extension strength *was* predictive of incident *symptomatic* (i.e., radiographic evidence plus symptoms) tibiofemoral OA. Out of 1,232 women and 846 men who did not have *symptomatic* tibiofemoral OA at baseline, 201 of 1,989 knees in women and 109 of 1,403 knees in men had incident *symptomatic* knee OA 30 months later. Knee extensor strength at baseline was 114 ± 41 N·m for men who developed *symptomatic* OA compared with 130 ± 42 N·m for those who did not (Figure 3). In women, knee extensor strength at baseline was 65 ± 26 N·m for those who developed *symptomatic* knee OA compared with 75 ± 25 N·m for those who did not (Figure 3). When women and men were grouped by tertiles of knee extensor strength, the odds ratios for incident *symptomatic* OA in women and men with the highest strength levels (compared with the lowest) were 0.7 (CI: 0.6,0.9) and 0.7 (CI: 0.5,0.9), respectively. Thus, the MOST study demonstrated that weak quadriceps strength was predictive of incident *symptomatic*, but not incident *radiographic*, tibiofemoral OA in both men and women.

#### Muscle strength and the progression of knee OA

Despite the finding that leg extensor strength was a determinant of incident *symptomatic* knee OA, it does not appear that strength influences the progression of OA. In a study of 111 women and 154 men with symptomatic knee OA who were followed for 30 months, there were no associations of quadriceps strength with the loss of cartilage at the tibiofemoral joint in either women or men, regardless of limb alignment [21]. Odds ratios in high- versus low-strength groups (women and men combined) for cartilage loss in the medial and lateral tibiofemoral compartments were 1.0 (CI: 0.5, 1.8) and 1.1 (CI: 0.5, 2.5), respectively. However, quadriceps strength was protective against cartilage loss in the lateral aspect of the patellofemoral joint (OR: 0.4; CI: 0.2, 0.9). Greater quadriceps strength was also associated with less knee pain and better physical function, but analyses were not conducted separately in women and men. Similarly, among 57 women and 25 men with radiographic evidence of knee OA, only 14 women and 3 men were classified as having progressive OA when evaluated 31.5 months later. Knee extensor strength at baseline did not differ between those with progressive versus non-progressive OA [22].

#### Muscle function and knee OA

Because the contribution of muscle activity to the stresses experienced by knee-joint cartilage depends on how the muscles are used during dynamic actions, some knee OA studies have compared patterns of muscle activity in men and women when they walk. In one such study, a subgroup of individuals (64.2 ± 7.4 yrs; n= 60, 33 women) from the MOST study who exhibited radiographic and symptomatic signs of knee OA produced 400-m walk times (215–537 s; 304 ± 62 s) that were associated with different muscle strength and gait characteristics for men and women [23]. The gait analysis comprised kinematic and ground reaction force measurements as participants walked at self-selected and controlled speeds along a 9-m walkway. Stepwise regression models indicated that some of the variability in walking speed (400-m walk times) for the men was explained by measures of the power produced in the sagittal plane by muscles that span the hip and ankle joints when walking at a moderate speed (0.89 m/s) (R^2^ = 0.32, *P* = 0.025). The 400-m walk times for men were also correlated with isokinetic strength of the knee extensors and flexors, but not the hip muscles (abduction, extension, flexion). In contrast, no measure of isokinetic strength for the knee (extension and flexion) or hip (abduction, extension, flexion) muscles was associated with 400-m walk times in women, but a significant amount of the variability in their 400-m walk times was explained by the torque and power about the hip (frontal plane) and knee (sagittal plane) joints when walking at the moderate speed (0.89 m/s) (R^2^ = 0.61, *P* = 0.003). Thus, the time taken to walk 400 m, which provides an index of the repetitive loading of the knee joint, was associated with different measures of strength and mechanical output (torque and power) for lower limb muscles of men and women with similar levels of knee OA.

In people with symptomatic OA, walking speed (400-m walk times) decreased with age for the men and with the WOMAC pain score for women, but not with the Physical Activity Scale for the Elderly for either sex [23]. There were also modest-to-strong correlations between 400-m walk times and an index of lower limb function, the summary performance score for Short Performance Physical Performance Battery (SPPB), and 2-min walk distance. Furthermore, the torque and power produced by the knee muscles of men during walking did not differ with the level of functional mobility (composite SPPB score) and was similar to the findings reported for men without symptomatic knee OA. In contrast, higher functioning women exhibited greater ankle and hip (frontal plane) muscle activity during walking than those who were less mobile [23]. The findings indicated that men and women with greater mobility relied more on an ankle strategy than a hip strategy when walking, whereas men with less mobility decreased the ankle strategy and women with less mobility increased the hip strategy. Taken together, the results are consistent with the conclusion that higher functioning individuals with knee OA tend to modulate the mechanical output at the ankle more during walking than those who have more severe signs of knee OA and are less mobile [24], and that the more mobile women with knee OA had greater control of hip muscle activity in the frontal plane compared with lower functioning women [25,26].

Relatively few studies have examined the predictive power of declines in muscle function, as reflected in tests of physical function, for the development of knee OA. Thorstensson et al. [27] followed 148 individuals (62 women; 35–54 yrs) with chronic knee pain for 5 yrs and found that the number of one-leg rises from a chair predicted the *development* of radiographic signs of knee OA (41/94 participants) (OR 2.6; 95% CI = 1.1–6.0). In contrast, *progression* of the radiographic signs in 29/54 participants was not associated with any measure of physical function (leg rises, balance, 300-m walk). Furthermore, there were no sex differences between any of these associations.

In summary, the data indicate that the strength of the knee extensors can predict the odds of developing *symptomatic*, but not *radiographic*, signs of knee OA in both men and women. Furthermore, the most mobile women with knee OA had greater function of the hip muscles in the frontal plane, whereas differences in mobility for both men and women were related to muscle function in the sagittal plane at the hip, knee, and ankle joints. It is not known, however, whether sex differences in muscle function either precede the development of knee OA or contribute to its progression. We identify several gaps in knowledge related to the potential influence of muscle function on the sex differences in knee OA (Table 3).

### Obesity

Forces transmitted through the knee joint during walking can exceed four times body weight [28]. Consequently, increases in body weight, without associated compensatory adaptations in knee joint anatomy (e.g. subchondral bone) and limb kinematics and kinetics during movement (e.g. reduced stride length and walking speed, changes in knee adduction moments), would increase the stresses and strains in the knee joint during walking. Based on these associations, it has been hypothesized that the primary mechanisms by which obesity modulates knee joint integrity are through increases in joint loading and alterations in gait mechanics [29-31].

#### Obesity as a predictor of the onset and progression of knee OA

A number of observational studies have identified obesity as a risk factor for knee OA, with an increased risk for women compared with men. Body mass index (BMI) is a significant and independent predictor of the onset and progression of knee OA [32,33] and this effect is stronger in women than in men [34]. In the Framingham study, the relative risk of developing knee OA in overweight individuals was 2.07 times greater for women and 1.51 times greater for men than for those individuals with the lowest body-weights [33]. In an investigation of 5,193 individuals from the first US National Health and Nutrition Examination Survey (HANES I), a life history of obesity increased the risk of developing knee OA later in life for women, whereas there was no such relation for men [35]. Similarly, the Genetics of Osteoarthritis and Lifestyle (GOAL) case–control study identified BMI as a factor that increased the risk of developing knee OA (OR 2.68), with the risk for knee OA being greater in women (OR 3.23) than in men (OR 2.20) [36].

However, an increase in physical activity, which presumably involved an increase in knee-joint loads, did not significantly increase or decrease the risk of developing knee OA in the Framingham cohort [37]. Interestingly, a measure of body shape, the waist-to-hip ratio, was independently associated with increased risk of hip but not knee OA in women [36], whereas the distribution of body fat was not related to either hip or knee OA in men [36]. However, obesity was associated with a younger age at the time of arthroplasty surgery independent of sex in a cohort of patients receiving total knee replacements [38] and the risk of developing lower limb OA was also greater in individuals who became overweight earlier in life. Obese children have altered gait patterns and knee kinematics compared with normal weight children that may lead to OA of the medial compartment of the knee due to increased medial compartment stresses resulting from greater peak internal knee-abduction moments [39].

#### Obesity and limb alignment

It has been hypothesized that the relation between BMI and knee OA may be mediated through changes in limb alignment suggesting that limb alignment is adaptable and a function of the biomechanical loading through the joint. Accordingly, BMI correlated with the severity of OA in knees with a varus alignment, but not those with a valgus alignment in a study of 292 individuals [40]. However, BMI also was significantly correlated with varus alignment and much of the variance in knee OA explained by BMI was also explained by varus alignment. In knees of 2,660 individuals from the MOST cohort without knee OA at baseline, the risk of developing radiographic signs of tibiofemoral OA at 30 months was significantly greater for overweight or obese subjects (defined by BMI) compared with normal-weight individuals, and this effect was not modified by limb alignment [41]. In this same study, however, subjects with OA at baseline exhibited no association between obesity and a worsening of the radiographic evidence of knee OA at 30 months when not accounting for limb alignment. When limb alignment was considered, obesity had no influence on OA progression in those individuals with baseline varus alignment, whereas BMI was significantly associated with OA progression among individuals with neutral or valgus alignment at baseline [41]. The data were not stratified by sex in these studies.

#### Obesity and meniscus damage

Increased joint loading due to obesity may also play a role in damage to the meniscus. Damage to or partial or complete removal of the meniscus is an established risk factor for knee OA [42-44]. Menisectomy (removal of the meniscus) results in altered load transmission through the knee joint and subsequent alterations in the stress and strain patterns in the knee cartilage tissue consistent with clinically observed patterns of cartilage damage [45,46]. In a study of 387 patients with meniscal tears, radial tears of the medial meniscus, which have been shown to result in a 25% increase in cartilage contact pressure and an increase in varus alignment compared to an intact knee [47], were associated with older age, females, and obesity [48]. Laberge et al. [49], in an analysis of MRI data of 137 individuals (45–55 years old) from the Osteoarthritis Initiative, found that the prevalence of meniscal lesions was 64%, with a higher prevalence of meniscal tears in men (36%) than women (13%). Laberge et al. [49] also reported a nearly four-fold increase in meniscal tears in obese individuals compared with normal weight individuals, although this result was not stratified by sex [49].

#### Obesity and systemic factors

Although obesity may contribute to the risk of knee OA by increasing the loads experienced by the joint, obesity has also been associated with an increased risk of hand OA [50]. However, these data are equivocal with respect to sex. Although women have a greater risk of developing hand OA than men [51,52], a study that included both men and women showed a significant association between body weight and hand OA in men but not women [53], whereas a separate study of women only demonstrated that body weight was a significant predictor of incident hand OA [50]. Increasing evidence suggests that metabolic factors related to obesity, now regarded as a low-grade systemic inflammatory disease, influence systemic levels of cytokines, which interact with mechanical factors in the development of OA [54-57]. Individuals with OA have higher concentrations of leptin in synovial fluid and these levels are significantly correlated with BMI [58]. In addition, joint levels of leptin are greater in women compared with men [59]. Consistent with this hypothesis, a cross-sectional study investigating the effect of body mass composition (proportion of fat vs. muscle mass) on OA of 153 healthy subjects ranging from normal weight to obese found that body fat mass was positively associated with increased bone marrow lesions and cartilage defects (both are features of early knee OA), whereas there was no significant relation with skeletal muscle mass [60] suggesting that fat and muscle mass have differential effects on the development and progression of knee OA. In addition, an increase in physical activity for individuals with a BMI above the median within the Framingham cohort, which would imply increased mechanical loading within the knee joint, did not significantly increase (or reduce) the risk of knee OA [37]. These associations remain to be compared between the two sexes.

These results indicate that obesity is a significant risk factor for the development of knee OA, and that the association is stronger for women than for men. Although limb alignment does not contribute to the influence of obesity on incident knee OA, BMI does contribute to the progression of knee OA but only in neutral and valgus limbs. The mechanism by which obesity modulates OA appears to involve more than adverse changes in the local mechanical environment, likely involving synergistic systemic effects such as increases in inflammatory cytokines or alterations in hormone levels (see subsequent manuscript entitled “Hormonal Modulation of Connective Tissue Homeostasis and Sex Differences in Risk for Osteoarthritis of the Knee”). These interactions have not yet been compared between men and women and represent a significant gap in our understanding of the role sex differences play in the incidence and progression of knee OA. We identify several gaps in knowledge related to the influence of obesity on the sex differences in knee OA (Table 3).

**Table 3:** Gaps in knowledge on the contributions of mechanical factors to sex differences in knee OA

**Limb Alignment**	
1.	Are there sex differences in the prevalence of unilateral and bilateral limb alignment?
2.	How does limb alignment change across the lifespan for men and women?
3.	Are there sex differences in the prevalence of limb malalignment between obese men and obese women?
Muscle Function	
1.	Can strength training attenuate the incidence and progression of knee OA and is the intervention more or less effective in women?
2.	Do observed sex differences in muscle function during walking among individuals with knee OA contribute to either the development or worsening of the disease?
3.	What are the magnitudes of the cartilage stresses associated with differences in the mechanical output of lower limb muscles observed in men and women with knee OA during walking?
Obesity	
1.	Does the differential influence of fat and muscle mass on the development and progression of knee OA differ for men and women?
2.	Do circulating levels of inflammatory markers predict the sex difference in the prevalence of knee OA among older adults?
3.	Do meniscal lesions occur more frequently in men or women?

### Conclusions

Contact stress in knee-joint cartilage is a significant predictor of developing symptoms that are interpreted to indicate the presence of knee OA. Limb alignment, as a surrogate measure that may modulate knee-joint mechanics, does not account for observed sex differences in the prevalence of knee OA. Although weakness of the knee extensor muscles is predictive of the incidence of symptomatic knee OA, the association is similar for men and women. Nonetheless, the mechanical output of lower limb muscles (torque and power at the hip, knee, and ankle) during walking differs for low- and high-functioning individuals, with the differences depending on the sex of the individual. However, it is not known whether or not the sex differences in muscle function during walking contribute to either the development or progression of knee OA. Although, obesity poses a greater risk for developing knee OA in women than men, the mechanism is unknown.

The structural integrity of the articulating surfaces within the knee joint depends on the microstructural organization and material properties of the cartilage and meniscus, the macroscopic structural morphology of the joint (e.g., articular surface shape, cartilage thickness, joint alignment, ligament morphology, meniscus size and shape), and the loads transmitted through the joint. There are redundant combinations of traits through which joint configurations can provide nominally equivalent functionality under normal loading conditions. These combinations can involve quite different sets of traits, and a subset of these combinations, although sufficient for everyday loading environments, may be suboptimal when subjected to slight perturbations in one or more traits or loading conditions. Consequently, the development and progression of knee OA can result from multiple, distinct combinations of numerous musculoskeletal and neuromuscular traits. However, the dominant study design in research on knee OA focuses on the role of one or a limited set of factors that may contribute to the disease. There are significant gaps in knowledge about how different combinations of musculoskeletal, morphological, metabolic, and biological traits synergistically combine to provide lifelong, robust, knee function. To identify the mechanisms responsible for sex differences in the initiation and progression of knee OA, it may be necessary to take a more integrative approach of examining the interactions among a greater number of potential factors than is typical in most studies on knee OA.

## Abbreviations

BMI: Body mass index; CI: Confidence interval; GOAL: Genetics of Osteoarthritis and Lifestyle; MOST: Multicenter Osteoarthritis Study; MRI: Magnetic resonance imaging; N: Newton; NHANES: National Health and Nutrition Examination Survey; OA: Osteoarthritis; OR: Odds ratio; SD: Standard deviation; SPPB: Short Performance Physical Performance Battery; WOMAC: Western Ontario and McMaster Universities Arthritis Index.

## Competing interests

The authors declare that they have no competing interests.

## Authors’ contributions

All authors contributed to the development of the review. All authors read and approved the final manuscript.
